# Shear bond strength and adhesive remnant index of metal brackets bonded after different post-bleaching waiting periods in enamel bleached with two bleaching agents: An in vitro study

**DOI:** 10.4317/jced.63892

**Published:** 2026-03-30

**Authors:** Angelo Miranda-Castro, Danny Ramírez-Tito, Leonor Castro-Ramirez, Luis Cervantes-Ganoza, Goretty Garcia-Luna, Marysela Ladera-Castañeda, César Cayo-Rojas

**Affiliations:** 1 School of Stomatology, Universidad Privada San Juan Bautista, Lima, Peru

## Abstract

**Background:**

Successful fixed orthodontic therapy using metal brackets depends on reliable bracket-to-enamel bonding. However, many patients undergo tooth bleaching before fixed orthodontics, which may alter enamel and compromise shear bond strength (SBS). The present in vitro study aimed to compare the SBS and the adhesive remnant index (ARI) of metal brackets bonded after different post-bleaching waiting periods to enamel bleached with two different agents.

**Material and Methods:**

An in vitro experimental study was performed using eighty-four human premolars extracted for orthodontic reasons and randomly allocated to seven groups: an unbleached control group and six experimental groups bleached with 22% carbamide peroxide or 35% hydrogen peroxide and evaluated after three post-bleaching intervals (24 hours, 7 days, and 14 days). Brackets were bonded using Orthocem. SBS was tested in a universal testing machine, and failure mode was assessed using the adhesive remnant index (ARI) under stereomicroscopy. SBS was analyzed using robust one-way ANOVA with Games-Howell post hoc comparisons (p &lt; 0.05), and ARI distributions were compared using Fisher's exact test (p &lt; 0.05).

**Results:**

SBS varied according to bleaching agent and post-bleaching interval (p &lt; 0.001). With 35% hydrogen peroxide, SBS peaked at 7 days (12.63 ± 4.26 MPa) and decreased at 14 days (4.84 ± 2.08 MPa) (p &lt; 0.05). With 22% carbamide peroxide, SBS also peaked at 7 days (9.73 ± 3.76 MPa) and decreased at 14 days (3.23 ± 1.46 MPa) (p &lt; 0.05). Between agents, SBS was similar at 24 hours and 7 days (p = 0.936 and p = 0.091) but higher with hydrogen peroxide at 14 days (p = 0.039). ARI distributions did not differ across intervals for either bleaching agent (p &gt; 0.05).

**Conclusions:**

The SBS of metal brackets depended on the type of bleaching agent and the time elapsed, suggesting that bleached enamel may behave variably during the immediate post-treatment period. In addition, after 14 days, a significant deterioration in adhesion was observed, especially with 22% carbamide peroxide, which could translate into a higher risk of detachment if cementation is performed at that time. On the other hand, the distribution of the IRA did not show significant changes over time, indicating that bleaching would mainly affect the magnitude of bond strength rather than the failure pattern. These findings support orthodontic planning that considers the bleaching agent used and the waiting time before cementation, although clinical studies are needed to confirm its applicability in patients.

## Introduction

Fixed orthodontic appliances contribute to the functional and aesthetic rehabilitation of occlusion, with a direct impact on the perception of the smile and quality of life related to oral health (1 , 2). The adhesion of brackets to enamel is a critical clinical element, as direct cementation must provide a stable bond without compromising the integrity of the enamel (3 , 4). Adhesion failures lead to increased treatment time, more appointments, and an increased risk of enamel damage associated with repeated debonding and recementation procedures (3 , 4). Likewise, aesthetic demands have increased the use of vital bleaching procedures before or during treatment with fixed orthodontic appliances. Commonly used protocols employ hydrogen peroxide in the office and carbamide peroxide for outpatient use with trays, showing significant variations in concentration, exposure time, and pH (5 , 6). In the field of orthodontics, several in vitro studies have reported a decrease in bond strength when bracket cementation is performed immediately after bleaching, although it has been reported that the magnitude of the effect varies depending on the agent, protocol, and adhesive system used (7 - 10). This can be attributed to peroxide and oxygen residues in the enamel, which are capable of inhibiting the polymerization of composite resins and altering the formation of the adhesive interface, which can lead to suboptimal bonding (11 - 13). Consequently, post-bleaching waiting intervals and/or oxidative neutralization strategies have been proposed as measures to optimize adhesion (12 , 13). Likewise, a recent systematic review, based mainly on in vitro studies, showed heterogeneity in agents, concentrations, and protocols, and reported the need to defer bracket cementation for approximately two weeks after bleaching in order to recover shear bond strength (14). However, uncertainty remains about the minimum clinically prudent time to cement brackets after bleaching, particularly when using concentrations commonly used in the office and high-concentration outpatient protocols (7 - 10 , 13). Additionally, the heterogeneity of experimental designs and adhesive systems limits comparability between studies (11 , 13). Therefore, it is necessary to provide comparative evidence on adhesive strength and failure patterns under controlled conditions with a standardized adhesive system on the combined impact of the bleaching agent and the waiting time for bracket cementation. Therefore, the present in vitro study aimed to compare the SBS and the adhesive remnant index (ARI) of metal brackets bonded after different post-bleaching waiting periods (24 h, 7 d, and 14 d) to enamel bleached with two different agents (35% hydrogen peroxide or 22% carbamide peroxide). The null hypotheses were: (i) for each bleaching agent, SBS does not differ among post-bleaching waiting periods (24 h, 7 d, and 14 d); and (ii) for each bleaching agent, ARI distributions do not differ among post-bleaching waiting periods.

## Material and Methods

**Figure 1:**
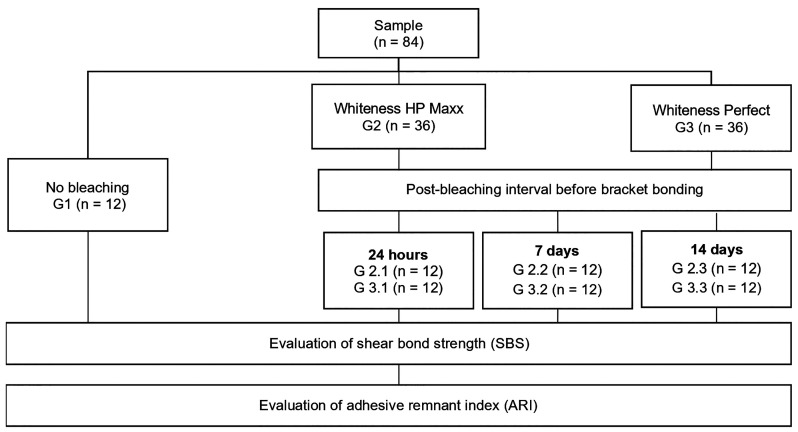
Random allocation of specimens in each group according to bleaching protocol and post-bleaching interval before bracket bonding.

1. Study design This in vitro experimental study was conducted at the School of Stomatology of the Universidad Privada San Juan Bautista and at the High Technology Laboratory Certificate (ISO/IEC Standard: 17025), Lima, Peru, between April and May 2025, with a letter of approval from the Institutional Research Ethics Committee No. 787-2025-CIEI-UPSJB. This study considered CRIS Guidelines (Checklist for Reporting In-vitro Studies) (15). 2. Sample calculation and selection Eighty-four permanent human premolars extracted for orthodontic reasons and collected within 3 months prior to the experiment were selected (4 , 16). An a priori sample size calculation was performed in G*Power (version 3.1.9.7) for a one-way ANOVA. The effect size (f = 3.02) was derived from a pilot study (n = 5/group) using the means and standard deviations of SBS. Assuming = 0.05 and power (1) = 0.80, the minimum sample size was 28 specimens. To account for expected variability in in vitro testing and to enable between-group comparisons, 12 specimens per group were included (N = 84). Specimens were randomly assigned to three main groups: G1, unbleached control (n = 12); G2, bleached with Whiteness HP Maxx (35% hydrogen peroxide; n = 36); and G3, bleached with Whiteness Perfect (22% carbamide peroxide; n = 36). Groups G2 and G3 were further randomized into three subgroups each (n = 12) according to the post-bleaching interval before bracket bonding (24 hours, 7 days, and 14 days) (Fig. 1).

1

Selection criteria: - Inclusion criteria: human premolars extracted for orthodontic reasons, collected no more than three months prior to the experiment. - Exclusion criteria: teeth with caries, fractures or enamel cracks, restorations on any surface, obvious discoloration or stains, enamel defects (e.g., hypoplasia or fluorosis), marked wear or abrasion on the vestibular surface, or any alteration that compromises the integrity of the enamel in the adhesion area. 3. Sample characteristics and preparation The teeth underwent initial cleaning to remove soft tissue and debris, and were immersed for one week in a 0.1% thymol solution to prevent bacterial proliferation and dehydration (16 , 17). They were then kept in distilled water at room temperature until the investigation was carried out. The water was changed every 24 hours, and the storage period did not exceed 3 months (16 , 17). 4. Tooth mounting and prophylaxis A heavy silicone condensation mold Zetaplus (Zhermack, Badia Polesine, Italy) with an internal diameter of 30 mm and a length of 30 mm was used (4 , 16). Fast-curing acrylic was poured into the mold and the teeth were placed vertically in it with the roots submerged and the longitudinal axis of the tooth kept parallel to the longitudinal axis of the mold (4 , 16). Prophylaxis was then performed with pumice stone using the EX-203C low-speed micromotor (NSK, Tokyo, Japan). The teeth were then rinsed for 10 seconds with a triple syringe and dried. 5. Bleaching protocol The control group (G1) did not undergo bleaching. The experimental groups (G2 and G3) were bleached with 35% hydrogen peroxide and 22% carbamide peroxide, respectively (Table 1).

Table 1

**Figure 2:**
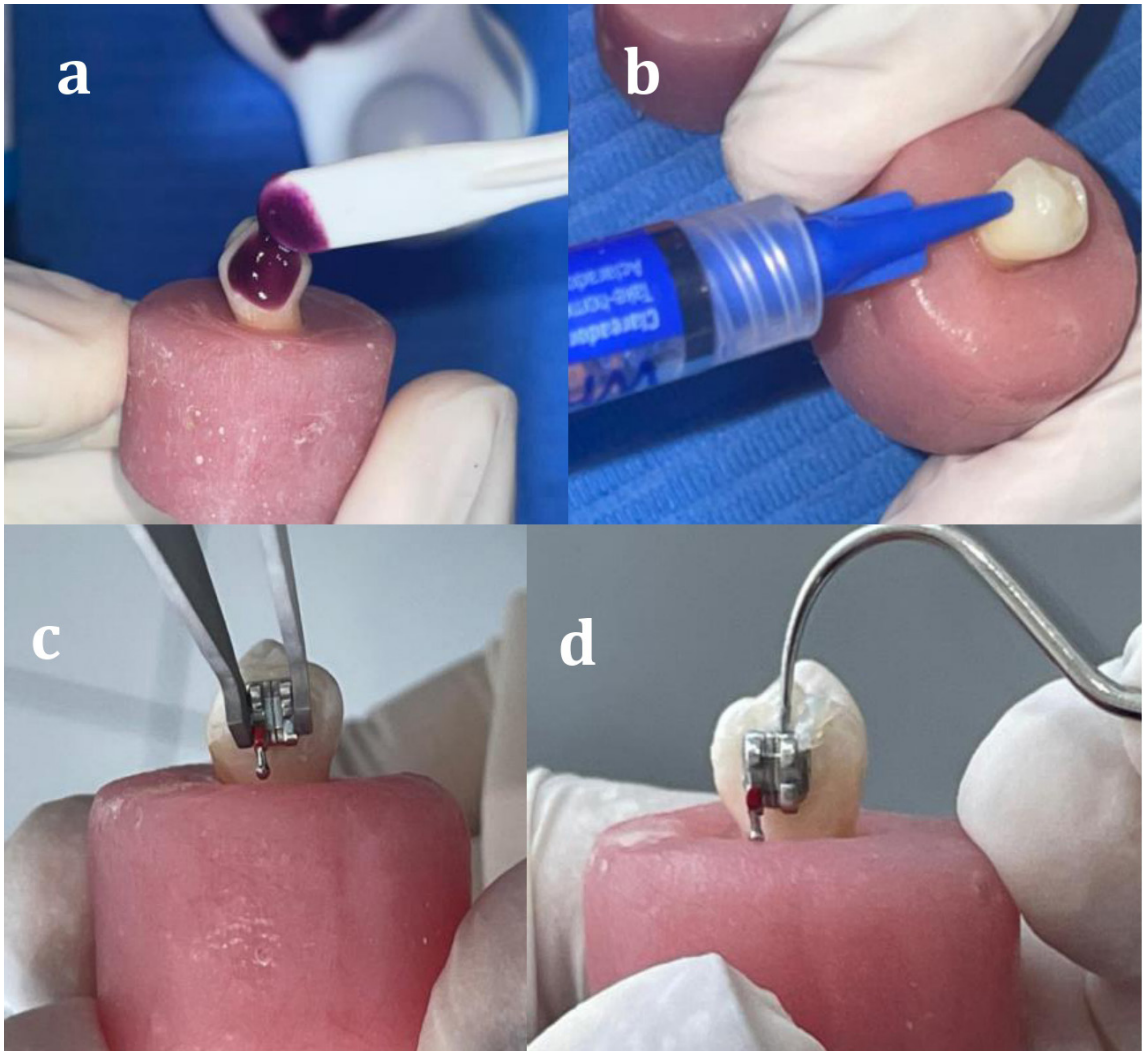
a) Application of Whiteness HP bleaching gel, b) Application of Whiteness Perfect bleaching gel, c) Placement of metal brackets with bracket forceps, d) Removal of excess gel with a dental explorer.

Bleaching procedures were performed according to the manufacturers' instructions (Fig. 2).

2

**Figure 3:**
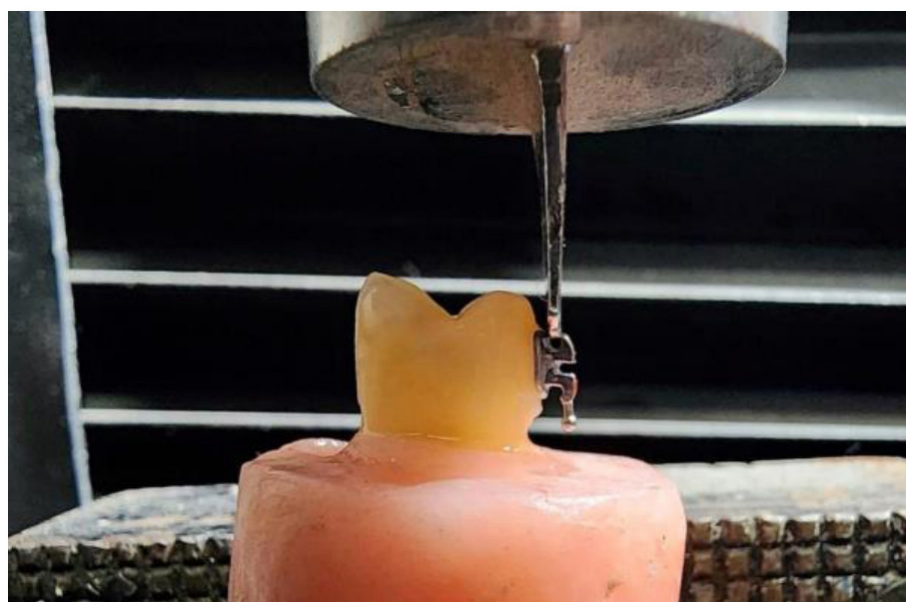
Shear bond strength test.

For group G2, Whiteness HP Maxx (35% hydrogen peroxide; FGM, Joinville, Brazil) was mixed (three drops of peroxide to one drop of thickener) and applied as a 1 mm-thick layer on the buccal enamel surface. The gel remained in place for 45 min per session, divided into three 15-min applications. After each session, the gel was removed with gauze and the specimens were rinsed with water and stored in distilled water at room temperature, renewed every 24 h, until the bonding procedure (17 , 18). For group G2, Whiteness HP Maxx (35% hydrogen peroxide; FGM, Joinville, Brazil) was mixed (three drops of peroxide to one drop of thickener) and applied as a 1 mm-thick layer on the buccal enamel surface. The gel remained in place for 45 min per session, divided into three 15-min applications. After each session, the gel was removed with gauze and the specimens were rinsed with water and stored in distilled water at room temperature, renewed every 24 h, until the bonding procedure (17 , 18). 6. Adhesion protocol For bracket bonding, the buccal enamel surface was etched with 37% phosphoric acid (Condac 37; FGM, Joinville, Brazil) for 15 seconds, rinsed for 10 seconds, and air-dried for 5 seconds. A light-cured adhesive (Ambar; FGM, Joinville, Brazil) was applied and actively rubbed for 10 seconds, then light-cured for 10 seconds (4 , 16) using an LED curing unit (iLed Plus; Woodpecker, Guilin, China) with an irradiance of 1000 mW/cm². Light-cured orthodontic resin cement (Orthocem; FGM, Joinville, Brazil) was placed on the bracket base, and metal premolar brackets with a mesh base (Azdent, Zhengzhou, China) were positioned at the center of the clinical crown. Excess resin was removed with a dental explorer, and the cement was light-cured according to the manufacturer's recommendations (4 , 16) (Fig. 2). The control group was cemented directly. In the experimental groups, bracket cementation was performed after a waiting period following bleaching of 24 hours (G2.1 and G3.1), 7 days (G2.2 and G3.2), and 14 days (G2.3 and G3.3). 7. Shear bond strength A universal testing machine (CMT5L, 7419, LG, Seoul, Korea) was used. The crosshead speed was set to 0.75 mm/min. A force was applied to the brackets in the occlusogingival direction using a blade until they detached. The values obtained in Newtons (N) were recorded on a data collection sheet and then converted to megapascals (MPa) by dividing the force (N) by the area of the bracket base (mm²) (4 , 16) (Fig. 3).

3

8. Evaluation of adhesive remnant A stereomicroscope (Leica EZ4, Wetzlar, Germany) with 10x magnification was used after a shear test to observe the vestibular surface of the tooth enamel (19 - 21). Failure mode scores were recorded according to the original description provided by Artun and Bergland (22), who classified them as value 0 (no adhesive left on the tooth), value 1 (less than half of the adhesive left on the tooth), value 2 (more than half of the adhesive left on the tooth), and value 3 (all adhesive left on the tooth with visible mesh pattern) (16 , 17 , 19 , 20). 9. Statistical analysis The data were imported into the statistical packages SPSS v24.0 and Stata v18.0 from a Microsoft Excel 2019 spreadsheet. Descriptive analysis of the quantitative variables was performed using the mean and standard deviation, while for the qualitative variables, tables of absolute and relative frequencies were prepared. For the inferential analysis, robust ANOVA with Games-Howell post hoc test was used for the comparison between groups, after verifying the assumptions of normality and homoscedasticity using the Shapiro-Wilk and Levene tests, respectively. To compare two groups, Student's t-test for independent samples was applied, also after verifying the statistical assumptions. The comparison of proportions of the ARI was performed using Fisher's exact test. The level of statistical significance was set at p &lt; 0.05.

## Results

In the analysis of shear strength (MPa) according to bleaching agent and evaluation time, it was observed that in the group treated with 35% hydrogen peroxide, the highest values were recorded at 7 days (12.63 ± 4.26 MPa) while the lowest value was recorded at 14 days (4.84 ± 2.08 MPa). Similarly, in the group treated with 22% carbamide peroxide, the highest values were recorded at 7 days (9.73 ± 3.76 MPa), while the lowest value was recorded at 14 days (3.23 ± 1.46 MPa). Statistically significant differences were observed in both types of bleaching (p &lt; 0.001) (Table 2).

Table 2

The Games-Howell post hoc analysis showed that, in the group with 35% hydrogen peroxide, shear bond strength was significantly higher at 7 days compared to the initial value (p = 0.008), at 24 hours (p = 0.024) and at 14 days (p &lt; 0.001), indicating a peak in strength at that point. In the group treated with 22% carbamide peroxide, a significant decrease was observed at 14 days compared to pre-treatment values (p = 0.001), at 24 hours (p &lt; 0.001), and at 7 days (p &lt; 0.001), suggesting a progressive loss of resistance after one week (Table 3).

Table 3

When comparing shear bond strength at 24 hours and 7 days, no significant differences were observed between 35% hydrogen peroxide and 22% carbamide peroxide (p = 0.936 and p = 0.091, respectively). However, at 14 days, a significant difference was observed in favor of 35% hydrogen peroxide (p = 0.039) (Table 4).

Table 4

**Figure 4:**
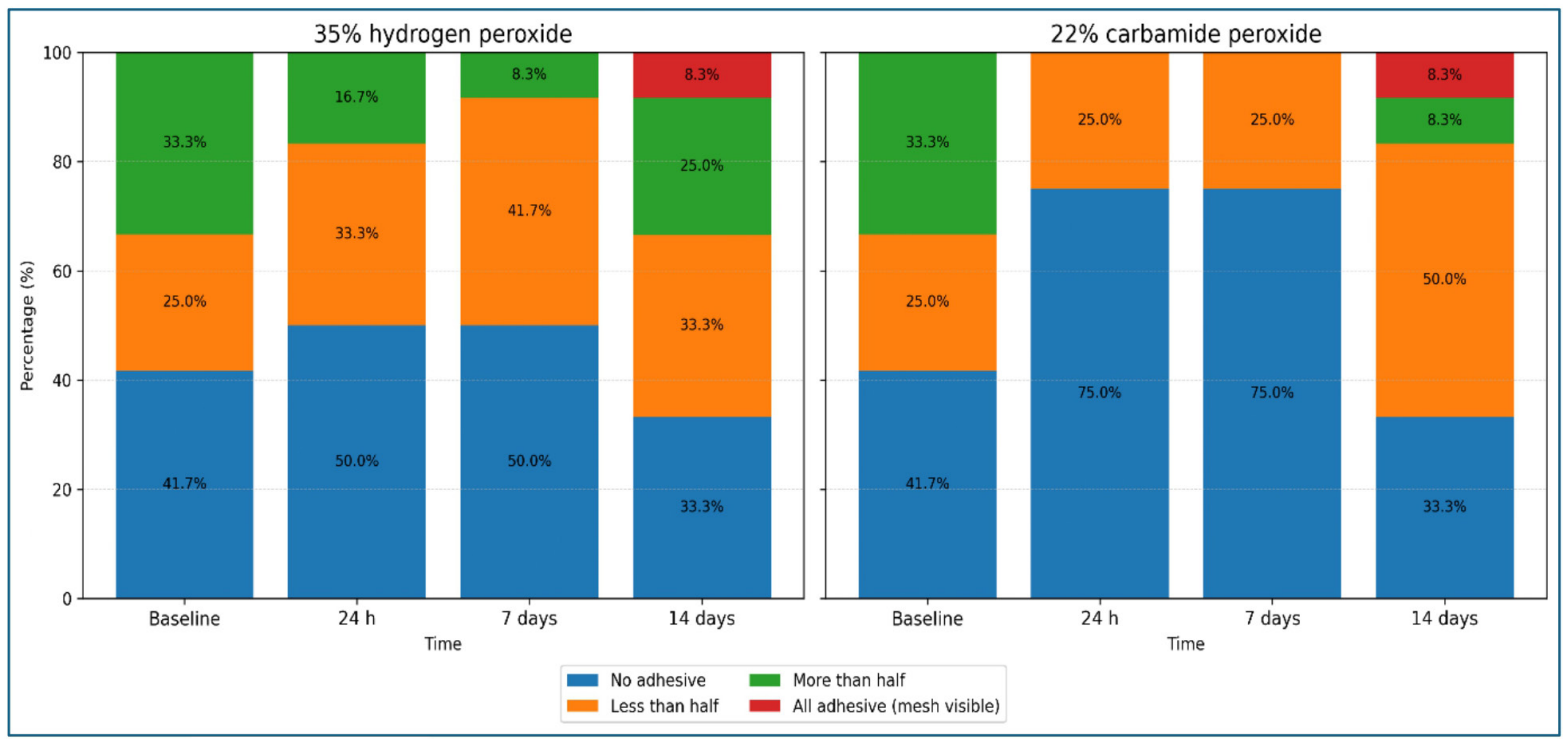
Distribution of ARI scores according to bleaching agent and post-bleaching interval.

Regarding ARI outcomes, approximately half of the teeth in the 35% hydrogen peroxide group showed ARI score 0 across all intervals (42-50%), and surfaces with more than 50% of adhesive remnant slowly decreased from 33% to 8% after 14 days. In the 22% carbamide peroxide group, the frequency of ARI score 0 increased at 24 hours and 7 days (75%) and decreased from 33% to 0% at 14 days (33%), whereas teeth with less than 50% of adhesive remnant decreased from 33% to 0% by 14 days. No significant differences in ARI score distributions were observed across post-bleaching intervals for 35% hydrogen peroxide (p = 0.847) or 22% carbamide peroxide (p = 0.067) (Table 5, Fig. 4).

Table 5

4

## Discussion

Many patients request tooth bleaching before initiating fixed orthodontic treatment (23). Because bleaching can modify the enamel surface and leave residual peroxide or oxygen species, bracket adhesion may be adversely affected (24). Accordingly, a delay between bleaching and bracket bonding has been recommended to allow recovery of bond strength (18). In the present study, SBS decreased significantly at 14 days for both bleaching agents. Therefore, first null hypothesis was rejected. The second null hypothesis was accepted, as ARI distributions did not differ significantly across intervals for either bleaching agent. Both bleaching protocols (22% carbamide peroxide and 35% hydrogen peroxide) produced significant changes in bond strength compared with the unbleached control, in agreement with previous reports from This finding coincides with that reported by De Almeida et al. (18) and Rojas et al. (23). This effect has been attributed to whitening agents that denature organic components of the enamel, increase porosity, and release free radicals; together, these mechanisms can induce chemical softening and/or degradation of resinous materials. Consequently, the integrity of the adhesive resin of the orthodontic cement is compromised, polymerization is inhibited, and the adhesion strength to the enamel is reduced (14 , 24). Baldión (25) points out that residual peroxide and/or oxygen released by whitening agents can interfere with the polymerization of adhesive systems and composite resins by altering the formation of the three-dimensional polymer network in methacrylate-based resins. This reduces the degree of conversion and, therefore, the adhesive strength. However, the magnitude of this effect may depend on the bleaching agent concentration and formulation. It was noticed that SBS peaked at 7 days for both bleaching agents and decreased at 14 days. Some authors have suggested that bonding around 7 days may be favorable because resin tag formation and penetration may resemble that of unbleached enamel (26 , 27). However, Almeida et al. reported different time-dependent patterns, including higher bond strength at 14 days (18), Nascimento et al. reported adequate values shortly after bleaching (28), and Bishara et al. (29) and Uysal et al. (30) reported no differences among waiting times. Methodological differences (e.g., lower bleaching concentrations, different adhesive systems, and storage media such as artificial saliva with remineralizing capacity) may partially explain these discrepancies (31 - 33). Although the synthesized evidence suggests that an interval of approximately two weeks after bleaching may be necessary to recover adhesive strength (14), these findings derive from heterogeneous protocols in terms of agent concentration, post-bleaching chronology, and storage conditions. In the present study, the lower SBS value at 14 days could be influenced by the definition of the waiting time (counted from the last application of the agent), the high concentration used (22% carbamide peroxide and 35% hydrogen peroxide), and storage in distilled water without ions with remineralizing potential. Consequently, a 14-day interval does not necessarily guarantee SBS recovery in all protocols, particularly under in vitro conditions without remineralizing capacity. On the other hand, the decrease in SBS at 14 days is probably due to the persistence of residual peroxide or oxygen and reactive species in the enamel which, even after two weeks, can interfere with the polymerization of the adhesive system and reduce the degree of conversion, weakening the resin-enamel interface (14 , 24 , 25). Additionally, microstructural changes induced by the oxidative process (e.g., in interprismatic regions) could compromise micromechanical retention and contribute to reduced bond strength to enamel (33). Regarding ARI, no significant differences were observed across post-bleaching intervals for either bleaching agent, although a higher frequency of ARI score 0 was observed after carbamide peroxide at 24 hours and 7 days, suggesting a greater tendency for adhesive failure at the enamel-resin interface. Carbamide peroxide decomposes into urea and hydrogen peroxide; subsequent degradation generates water and oxygen, and urea breakdown can increase pH via ammonia production (34 , 35). In addition, Carbamide peroxide gels also usually have a longer-lasting impact, while hydrogen peroxide treatments in the office try to work faster (36). Likewise, in gel formulations (such as Whiteness HP Maxx - 35% hydrogen peroxide), the use of thickeners such as carbopol increases viscosity, improves the permanence of the material on the tooth surface, and can prolong the release of reactive species. Furthermore, it should be noted that carbopol may be associated with a more acidic pH and surface changes in the enamel, which may explain greater adhesive retention (33). Regarding the adhesion values obtained, the literature indicates that, from a clinical point of view, SBS values in the range of 6 to 8 MPa are considered acceptable (14 , 37). In the present study, the initial values were within this range. After bleaching, at 24 hours and 7 days of waiting for bracket cementation, the values even increased, suggesting an initially stronger bond. However, after 14 days of waiting, a decrease below 6 MPa was observed, so that the minimum threshold considered clinically acceptable was no longer reached. In practical terms, this would indicate that bleaching may compromise enamel adhesion after a 14-day waiting period for bracket bonding, so it would not be advisable to perform adhesive procedures during that period due to the risk of insufficient bonding. These findings are clinically relevant because bleaching is increasingly performed before or during orthodontic treatment. Bleaching can temporarily alter enamel structure and release residual oxygen, which may compromise resin bonding and increase the risk of bracket detachment (38). The present study provides experimental evidence that could guide the most appropriate timing for placing braces after whitening, by analyzing different intervals between whitening and cementation, as well as comparing two widely used whitening agents. However, clinical extrapolation should be interpreted with caution and requires confirmation through in vivo studies and randomized clinical trials. A strength of this study was the use of distilled water as the storage medium, minimizing the influence of remineralizing ions and allowing the observed SBS changes to be primarily attributed to bleaching and the adhesive system (14 , 37). Another strength is the inclusion of two whitening agents (22% carbamide peroxide and 35% hydrogen peroxide), for which the available evidence regarding optimal waiting times before bonding is still limited. A detailed failure mode assessment was also performed, which allowed the damage to the enamel after bracket removal to be identified and described, providing valuable information for future clinical trials. One limitation of the present study was that, as it was an in vitro investigation, the results did not fully reproduce the actual conditions of the oral cavity. Factors such as pH, the presence of saliva and enzymes, temperature changes, food intake, and masticatory forces can directly influence the adhesive strength and long-term clinical performance of the materials and cannot be fully simulated in a laboratory setting. Another limiting factor is the methodological heterogeneity among the studies reviewed, especially in the way the experimental groups were formed, the concentrations of the bleaching agents, and the way the results were reported, which makes direct comparison and uniform conclusions difficult. Future studies should assess strategies to shorten the post-bleaching waiting period, such as enzymatic (catalase, peroxidase) or non-enzymatic antioxidants (e.g., sodium ascorbate), as well as ethanol, sodium bicarbonate, or vitamin E, which have been proposed to neutralize residual oxidants and restore bond strength (25). It is also suggested to evaluate the type of bracket mesh, as well as to evaluate different types of adhesives available on the market. This is relevant due to the diversity of solvents used in their composition, including alcohol and acetone. It is also recommended to determine which of these adhesives offers the lowest risk of bonding failures, in order to optimize the effectiveness and reliability of the bonding system.

## Conclusions

The results of this in vitro study showed that the shear bond strength of orthodontic brackets cemented to enamel subjected to teeth bleaching is influenced by the type of agent used and the time elapsed. 35% hydrogen peroxide reached its highest shear bond strength at 7 days, while 22% carbamide peroxide showed a progressive decrease, with the lowest values at 14 days. Although both agents showed similar behavior in the short term, after 14 days hydrogen peroxide showed significantly superior results. In addition, carbamide peroxide favored greater adhesive removal in the first 24 hours and up to 7 days. The distribution of the adhesive remnant index did not vary significantly over time. These findings highlight the importance of considering the type of bleaching agent and the time elapsed before orthodontic cementation. Future clinical studies are needed to confirm these results.

## Figures and Tables

**Table 1:** Technical profile of bleaching agents used.

Product	Composition	Manufacturer	Lot
Whiteness HP Maxx	35% hydrogen peroxide, thickener, neutralizer, glycol, and water	FGM, dental products Joinville, Brazil	031224130125191124
Whiteness Perfect	22% carbamide peroxide, 0.50% potassium nitrate, and 0.25% sodium fluoride	FGM, dental products Joinville, Brazil	150125

1

**Table 2:** Descriptive statistics and means comparison of SBS (MPa) according to post-bleaching interval and bleaching agent.

Bleaching agent	Time	n	Mean	SD	SE	95% CI		p*	p**	p***
						LL	UL			
35% Hydrogen peroxide	Before	12	7.33	2.54	0.73	5.71	8.94	0.424	0.030	<0.001***
	24 hours	12	9.56	4.55	1.31	6.67	12.45	0.061		
	7 days	12	12.63	4.26	1.23	9.92	15.33	0.741		
	14 days	12	4.84	2.08	0.60	3.52	6.16	0.414		
22% Carbamide peroxide	Before	12	7.33	2.54	0.73	5.71	8.94	0.424	0.035	<0.001***
	24 hours	12	9.69	3.55	1.03	7.43	11.95	0.474		
	7 days	12	9.73	3.76	1.09	7.34	12.12	0.134		
	14 days	12	3.23	1.46	0.42	2.30	4.16	0.221		

n: sample size; SD: standard deviation; SE: standard error of the mean; 95% CI: 95% confidence interval, LL: lower limit, UL: upper limit. *Based on the Shapiro-Wilk normality test (p>0.05, normal distribution). **Based on Levene’s homoscedasticity test (p>0.05, homogeneous variances). ***Based on robust one-way ANOVA (p<0.05, significant differences).

**Table 3:** Pairwise comparisons of SBS (MPa) among post-bleaching intervals for each bleaching agent.

Bleaching agent	Time	Time		
		24 hours	7 days	14 days
35% Hydrogen peroxide	Before	p = 0.469	* p = 0.008	p = 0.070
	24 hours		p = 0.345	* p = 0.024
	7 days			* p < 0.001
22% carbamide peroxide	Before	p = 0.270	p = 0.288	* p = 0.001
	24 hours		p = 1.000	* p < 0.001
	7 days			* p < 0.001

* Based on Games-Howell post hoc (p<0.05, significant differences).

**Table 4:** Comparison of SBS (MPa) between bleaching agents at each post-bleaching interval.

Time	Bleaching agent	n	Mean	SD	SE	95% CI		p*	p**	p***
						LL	UL			
24 hours	35% Hydrogen peroxide	12	9.56	4.55	1.31	6.67	12.45	0.061	0.321	0.936
	22% Carbamide peroxide	12	9.69	3.55	1.03	7.43	11.95	0.474		
7 days	35% Hydrogen peroxide	12	12.63	4.26	1.23	9.92	15.33	0.741	0.690	0.091
	22% Carbamide peroxide	12	9.73	3.76	1.09	7.34	12.12	0.134		
14 days	35% Hydrogen peroxide	12	4.84	2.08	0.60	3.52	6.16	0.414	0.188	0.039***
	22% Carbamide peroxide	12	3.23	1.46	0.42	2.30	4.16	0.221		

n: sample size; SD: standard deviation; SE: standard error of the mean; 95% CI: 95% confidence interval, LL: lower limit, UL: upper limit. *Based on the Shapiro-Wilk normality test (p>0.05, normal distribution). **Based on Levene’s homoscedasticity test (p>0.05, homogeneous variances). ***Based on Student’s t-test for independent samples (p<0.05, significant differences).

**Table 5:** Distribution of ARI scores according to bleaching agent and post-bleaching interval.

Bleaching agent	ARI	Time				p*
		Before	24 hours	7 days	14 days	
35% hydrogen peroxide	No adhesive on tooth	5 (41.7)	6 (50.0)	6 (50.0)	4 (33.3)	0.847
	Less than half of the adhesive on tooth	3 (25.0)	4 (33.0)	5 (41.7)	4 (33.3)	
	More than half of the adhesive on tooth	4 (33.3)	2 (16.7)	1 (8.3)	3 (25.0)	
	All adhesive on tooth with visible mesh pattern	0 (0.0)	0 (0.0)	0 (0.0)	1 (8.3)	
22% carbamide peroxide	No adhesive on tooth	5 (41.7)	9 (75.0)	9 (75.0)	4 (33.3)	0.067
	Less than half of the adhesive on tooth	3 (25.0)	3 (25.0)	3 (25.0)	6 (50.0)	
	More than half of the adhesive on tooth	4 (33.3)	0 (0.0)	0 (0.0)	1 (8.3)	
	All adhesive on tooth with visible mesh pattern	0 (0.0)	0 (0.0)	0 (0.0)	1 (8.3)	

ARI: Adhesive Remnant Index. *Based on Fisher’s exact test (p<0.05, significant differences).

## Data Availability

The datasets used and/or analyzed during the current study are available from the corresponding author.
